# Remote Co-Loading of Doxorubicin and Hydralazine into PEGylated Liposomes: In Vitro Anti-Proliferative Effect Against Breast Cancer

**DOI:** 10.3390/molecules30071549

**Published:** 2025-03-31

**Authors:** Walhan Alshaer, Zainab Lafi, Hamdi Nsairat, Baidaa AlQuaissi, Dana A. Alqudah, Hadil Zureigat, Islam Hamad

**Affiliations:** 1 Cell Therapy Center, The University of Jordan, Amman 11942, Jordan; walhan.alshaer@ju.edu.jo (W.A.); b.alquaissi@ju.edu.jo (B.A.); d_alqudah@ju.edu.jo (D.A.A.); 2 Pharmacological and Diagnostic Research Center, Faculty of Pharmacy, Al-Ahliyya Amman University, Amman 19328, Jordan; h.alnseirat@ammanu.edu.jo; 3 Department of Internal Medicine, Cleveland Clinic, Cleveland, OH 44195, USA; hadilzuri@gmail.com; 4 Faculty of Health Sciences, American University of Madaba, Amman 11821, Jordan

**Keywords:** remote loading, pH gradient, breast cancer, combination therapy, cardiotoxicity, liposomes, doxorubicin, hydralazine

## Abstract

Doxorubicin (DOX), an anthracycline chemotherapeutic agent, demonstrates efficacy against various types of cancer. Combining DOX with the antihypertensive drug hydralazine (HDZ) has been proposed as cardioprotective combination therapy, allowing for the use of a reduced DOX dose. The current study describes the remote co-loading of DOX and HDZ into PEGylated liposomes using, for the first time, a simultaneous pH gradient technique. First, PEGylated liposomes were prepared using an ethanol injection method and remotely loaded with DOX and HDZ. Then, DOX- and HDZ-loaded liposomes (Lip-DOX-HDZ) were characterized using DLS, TEM, FTIR, thermal analysis, drug leakage, and stability. Furthermore, the cellular uptake and cytotoxicity were evaluated in two human breast cancer cell lines (MCF7 and MDA-MB-231) and two normal cell lines (human dermal fibroblasts (HDFs) and rat cardiac cells (H9C2)). The results revealed that Lip-DOX-HDZ had a particle size of 158 ± 18 nm, PDI of 0.22 ± 0.08, and zeta potential of −22 ± 5 mV. The encapsulation efficiency of DOX and HDZ was 90% and 30%, respectively. Moreover, the IC_50_ values of Lip-DOX-HDZ showed higher cytotoxicity against the MDA-MB-231 (5.5 ± 0.4 µM) and MCF7 (6.25 ± 0.9 µM) breast cancer cell lines compared to normal cells: HDF cells (20 ± 3.0 µM) and H9C2 cardiac cells (19.37 ± 2.0 µM). Our study found that remotely loaded Lip-DOX-HDZ showed a ~4-fold lower toxicity and selectivity for normal cells (HDFs and H9C2), compared to breast cancer cells. This suggests that Lip-DOX-HDZ is a promising nanocarrier for both DOX and HDZ, clinically potent molecules.

## 1. Introduction

Doxorubicin (DOX), an anthracycline chemotherapeutic agent, is widely recognized for its effectiveness against various cancer types [1,2,3]. Its cytotoxic effects are mediated through multiple mechanisms, including DNA intercalation, topoisomerase II inhibition, interference with mitochondrial function, and free radical formation, all contributing to oxidative damage and DOX’s anti-cancer properties [4]. Unfortunately, cancer cells can develop resistance to DOX through genetic mutations and adaptive changes, leading to treatment failure and cancer recurrence [5,6,7,8]. Additionally, the dosage of DOX is limited by its cardiotoxicity [9], which can be either reversible (dose-independent) or irreversible (dose-dependent) [10]. Numerous strategies have been developed to enhance cancer cells’ sensitivity to DOX while reducing its cardiotoxic effects [11,12]. Combination therapy has emerged as a promising approach, offering improved treatment outcomes with fewer side effects [13]. Combining DOX with other drugs of lower toxicity, such as immunomodulatory agents or epigenetic targeting agents, has gained significant attention [14].

Among the various DOX formulations, PEGylated liposome-encapsulated DOX, such as Doxil or Caelyx, has shown several advantages [15,16]. These include enhanced targeting through the enhanced permeability and retention (EPR) effect [17], prolonged circulation time, increased drug accumulation at tumor sites, and reduced cardiotoxicity due to minimized exposure of cardiac tissues to the drug [18]. Therefore, a thorough understanding of the unique attributes of liposomal DOX and its comparison with other formulations is crucial for optimizing cancer treatment strategies [19].

Hydralazine (HDZ), which is a vasodilator used for the management of hypertension, has been repurposed as an adjuvant in chemotherapy due to its ability to modulate cancer cells epigenetically [20,21,22,23]. HDZ decreases arterial resistance, improves blood flow, and reduces the cardiac workload [20,24]. Importantly, HDZ has been shown to reduce the expression of the DNA methyltransferases DNMT1 and DNMT3 [20]. These enzymes promote carcinogenesis by silencing tumor suppressor genes through hypermethylation [20]. Thus, HDZ can induce the re-expression of tumor suppressor genes, restoring cells’ sensitivity to chemotherapy [25,26].

Accumulating evidence from experimental studies has demonstrated HDZ’s benefits beyond its vasodilatory effects [27,28]. HDZ exhibits antioxidative, anti-apoptotic, and HIF-1α stabilization effects, promoting angiogenesis and vascular protection in normal cells [29,30]. Additionally, its renoprotective properties have been linked to its ability to induce DNA demethylation, along with its antioxidative and anti-inflammatory capabilities. Notably, HDZ has been shown to induce cell death in human p53-mutant leukemic T cells by activating the mitochondrial apoptotic pathway, with a concomitant increase in reactive oxygen species (ROS) [29]. Clinical studies have further demonstrated HDZ’s demethylating effects in breast cancer patients [31], with normotensive individuals safely tolerating HDZ at doses up to 200 mg/day alongside standard chemotherapy. The cardioprotective effects of HDZ are attributed to its antioxidative activity, DNA demethylation, anti-inflammatory effects, and inhibition of mitochondrial fission.

PEGylated liposome-encapsulated drugs carry several advantages, such as enhanced targeting through the enhanced permeability and retention (EPR) effect, prolonged circulation time, increased drug accumulation at tumor sites, and reduced cardiotoxicity due to minimized exposure of cardiac tissues to the drug. In a study by Chen et al. (2019), hydralazine was formulated in liposomes to enhance the penetration of DOX–liposome nanoparticles into hypoxic tumors [32]. PEGylated liposomes were prepared for the co-delivery of doxorubicin and hydralazine for breast cancer treatment. This was the first study co-encapsulating these two drugs into liposomes using remote loading techniques, with DOX and HDZ serving as model drugs. DOX was successfully encapsulated into the core of liposomes using a pH gradient technique. Therefore, a thorough understanding of the unique attributes of liposomal DOX and its comparison with other formulations is crucial for optimizing cancer treatment strategies [15,16,19]. 

**Figure 1:**
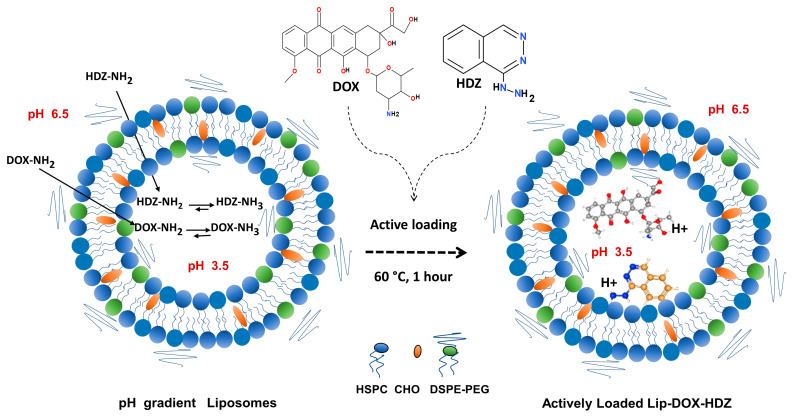
Schematic illustration of remote loading technique for DOX and HDZ together into pH gradient PEGylated liposomes.

In this study, we hypothesize that the remote co-loading of doxorubicin (DOX) and hydralazine (HDZ) into PEGylated liposomes represents a novel and effective formulation strategy to encapsulate multiple drugs into liposomes (Figure 1). This strategy is designed to offer a practical, simple method for the maximum encapsulation of drug combinations that improves the drug delivery and therapeutic efficacy of these drugs. Moreover, we propose that combining the known epigenetic modulation ability of HDZ [33,34,35] with the cytotoxic effects of DOX chemotherapy will act synergistically against tumor cells in breast cancer. Additionally, combining HDZ and DOX may improve therapeutic outcomes by reducing drug resistance in tumor cells and mitigating DOX-induced cardiotoxicity [36]. To the best of our knowledge, this is the first study to utilize the remote co-loading technique to encapsulate HDZ and DOX into liposomes, providing an important approach in developing effective drug delivery formulations in cancer therapy.

## 2. Results and Discussion

### 2.1. Liposomes’ Characterization and In Vitro Release

**Figure 2:**
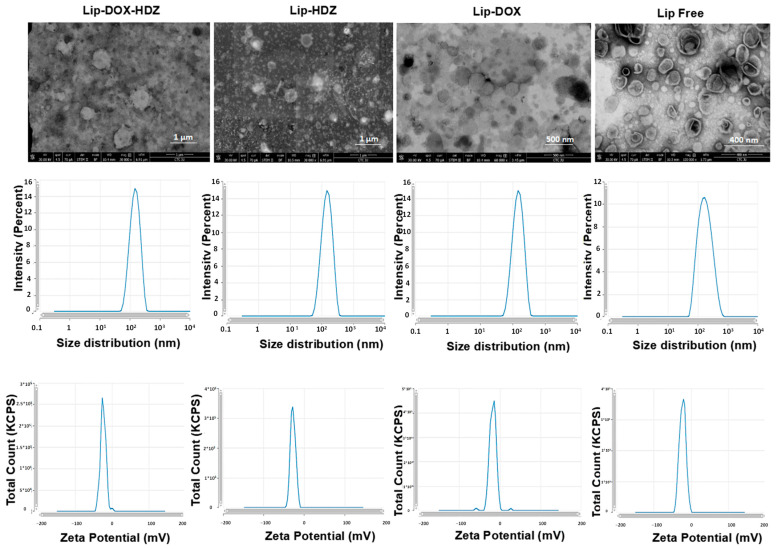
Liposomes’ characterization by TEM imaging, intensity size distribution by DLS, and average zeta potential.

DOX and HDZ were remotely encapsulated into PEGylated liposomes to maximize their anti-cancer activity and reduce side effects. The two drugs, DOX and HDZ, were successfully encapsulated into the liposomes by an active loading strategy using a pH gradient technique. The prepared liposomes exhibited good size and stability (Figure 2), with an average hydrodynamic diameter of 158 ± 18 nm, PDI of 0.22 ± 0.08, and zeta potential of −22 ± 5 (Table 1). Moreover, the TEM images in Figure 2 show that most of the liposomes ranged between 100 and 200 nm, which aligns with the DLS measurements. It is important to note that TEM generally provides smaller size estimates, as it measures the particle diameter, whereas DLS determines the hydrodynamic diameter, including the hydration layer surrounding the liposomes. HPLC-based cholesterol quantification revealed full recovery (~100%) of lipids after liposome formulation, indicating no lipid loss after liposome preparation and remote loading.

**Figure 3:**
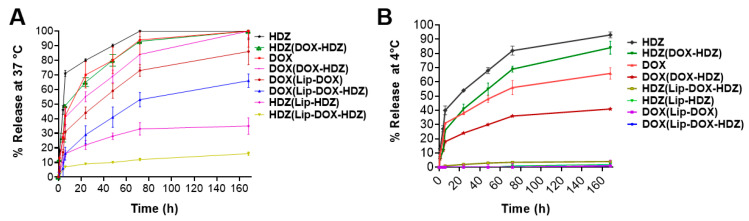
In vitro release kinetics: (**A**) In vitro release of DOX and HDZ as free drugs (individually or in combination) and from different liposomal formulations at 37 °C. (**B**) In vitro release of DOX and HDZ as free drugs (individually or in combination) and from different liposomal formulations at 4 °C. In this figure, HDZ in HDZ (DOX-HDZ), HDZ (Lip-HDZ), and HDZ (Lip-DOX-HDZ) refers to the release of HDZ. Similarly, DOX in DOX (DOX-HDZ), DOX (Lip-DOX), and DOX (Lip-DOX-HDZ) refers to the release of DOX. Data are presented as the mean ± SD (*n* = 3).

The encapsulation efficiency of DOX was 90%, and that of HDZ was 30%. The release characteristics of DOX and HDZ from liposomal and powder formulations were investigated to assess their release kinetics and differences in release behavior. Figure 3A,B represent the release kinetics at 4 °C and 37 °C, at a pH of 7.4. At both temperatures, the two drugs exhibited a higher release profile in their free form compared to their liposomal formulations. HDZ showed the highest release, reaching 80% at 4 °C and 70–95% at 37 °C after 72 h. On the other hand, DOX displayed lower release, at 35–55% from the physical mix (DOX-HDZ) at 4 °C and 70–90% from the physical mix (DOX-HDZ) at 37 °C after 72 h. Additionally, all liposomal formulations showed minimal leakage of the two drugs at storage temperature (4 °C) and medium release kinetics at physiological temperature (37 °C), with the lowest release for HDZ from the Lip-DOX-HDZ formula. The release behavior of HDZ from the co-loaded liposomes at 37 °C can be attributed to attractive forces between the two drugs, as both are trapped in the core of the liposomes, and DOX has a higher EE% than HDZ. The release kinetics of DOX from the liposomal formulation followed a biphasic pattern, with a relatively rapid release over the first 8 h, followed by sustained release. Several studies have investigated the release kinetics of DOX from Doxil using dialysis-based in vitro release assays, and they also supported the above release pattern [15,37,38]. They found that the release profile of doxorubicin from Doxil exhibited a biphasic pattern, with an initial burst release followed by a sustained release phase.

### 2.2. Thermogravimetric Analysis (TGA) and Differential Scanning Calorimetry (DSC)

**Figure 4:**
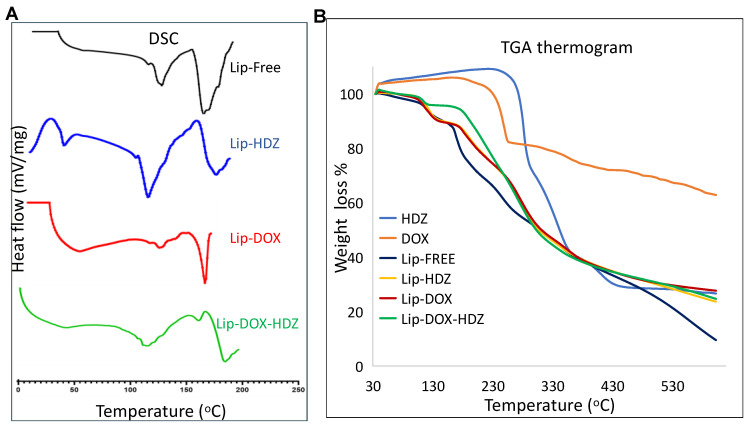
Thermal analysis of liposomal formulations encapsulated with DOX, HDZ, and DOX-HDZ: (**A**) Differential scanning calorimetry (DSC). (**B**) Thermogravimetric analysis (TGA).

The thermal stability of Lipo-DOX-HDZ, Lipo-DOX, and Lipo-HDZ was studied using TGA compared to free liposomes and free drugs in the temperature range of 35–600 °C. Figure 4B presents the thermograms of the weight loss curves for all samples. Free HDZ, free DOX, and free liposomes started to decompose at 220 °C, 210 °C, and 120 °C, respectively. DOX exhibited a major decrease in weight at 280 °C, while liposomes exhibited a decrease in weight in more than three major stages. The thermal change occurred between 100 °C and 120 °C in different liposome preparations due to the endothermic behavior that corresponded to the loss of water molecules from the liposomes, while the other stages were due to decomposition of the liposome components. Moreover, the TGA thermograms show a 65% weight loss and slight degradation of DOX at 600 °C, and almost complete degradation with 90% weight loss in the free HDZ and liposomes preparations. In addition, the drug encapsulation into liposomes displayed a slight enhancement, with no significant effect on the thermal decomposition behavior compared to free liposomes. This may be attributed to the drug loading percentages of free drugs. Accordingly, the thermal study of all liposomal formulations indicated a fairly stable formula construction at a transitional temperature of 200 °C, which remained intact until 220 °C [39].

The DSC method was used to determine the phase changes in the melting temperature of the liposomes due to the encapsulation of DOX and/or HDZ. Figure 4A represents the DSC endothermic peak of the free liposomes, Lip-DOX, Lip-HDZ, and Lipo-DOX-HDZ. The free liposomes showed two endothermic peaks at 135 and 175 °C. A slight shift in these peaks was observed for Lip-HDZ, at 125 and 185 °C, which may confirm the encapsulation of HDZ with low efficiency. On the other hand, Lipo-DOX showed an individual endothermic, low-intensity, and broad peak at 135 °C and a sharp, high-intensity peak at 170 °C. The significant differences in peak shapes can be attributed to the high efficiency of DOX encapsulation. Finally, there was a progressive broadening of the transition peaks, accompanied by a decrease in the transition phase, due to the encapsulation of both drugs in Lipo-DOX-HDZ.

The DSC thermograms showed exothermic peaks at 25 °C and 160 °C for Lipo-HDZ, suggesting different thermal behavior related to the liposomal integrity and interactions between HDZ and the lipid bilayer. Since HDZ has a lower EE%, some HDZ may interact with the lipid phase in a less stable or less ordered manner, leading to lipid reorganization and causing the exothermic reaction observed at 25 °C. Moreover, phase separation or crystallization of HDZ due to the presence of HDZ may have caused the second exothermic peak at 160 °C.

The slight shift in Lip-HDZ and the broadening of peaks in Lipo-DOX-HDZ suggest changes in lipid phase transitions that could affect liposome stability and drug release. The broadening in Lipo-DOX-HDZ indicates that both drugs may change the lipid bilayer’s organization, altering its permeability.

DOX has 90% EE%, contributing to sharper DSC peaks at 170 °C for Lipo-DOX. This high efficiency indicates stable DOX–liposome interactions, leading to a more organized lipid phase and controlled release, aligning with its biphasic release pattern at 4 °C and 37 °C. HDZ, with 30% EE %, shows less stable liposomal structures, as evident in the DSC peaks at 125 °C and 185 °C for Lip-HDZ. This reduced EE% is supported by HDZ’s higher release profile, indicating weaker entrapment and more rapid release.

Co-encapsulation of DOX and HDZ in Lipo-DOX-HDZ resulted in a complex release profile. The broadening and shifting peaks in the DSC data reflect interactions between the drugs and the lipid bilayer. The release of HDZ from Lipo-DOX-HDZ at 37 °C is likely influenced by attractive forces between DOX and HDZ, with DOX’s higher EE% potentially stabilizing the liposome and reducing HDZ’s release, as seen in the lower release of HDZ from the co-loaded liposomes compared to free HDZ.

### 2.3. FTIR Characterization

**Figure 5:**
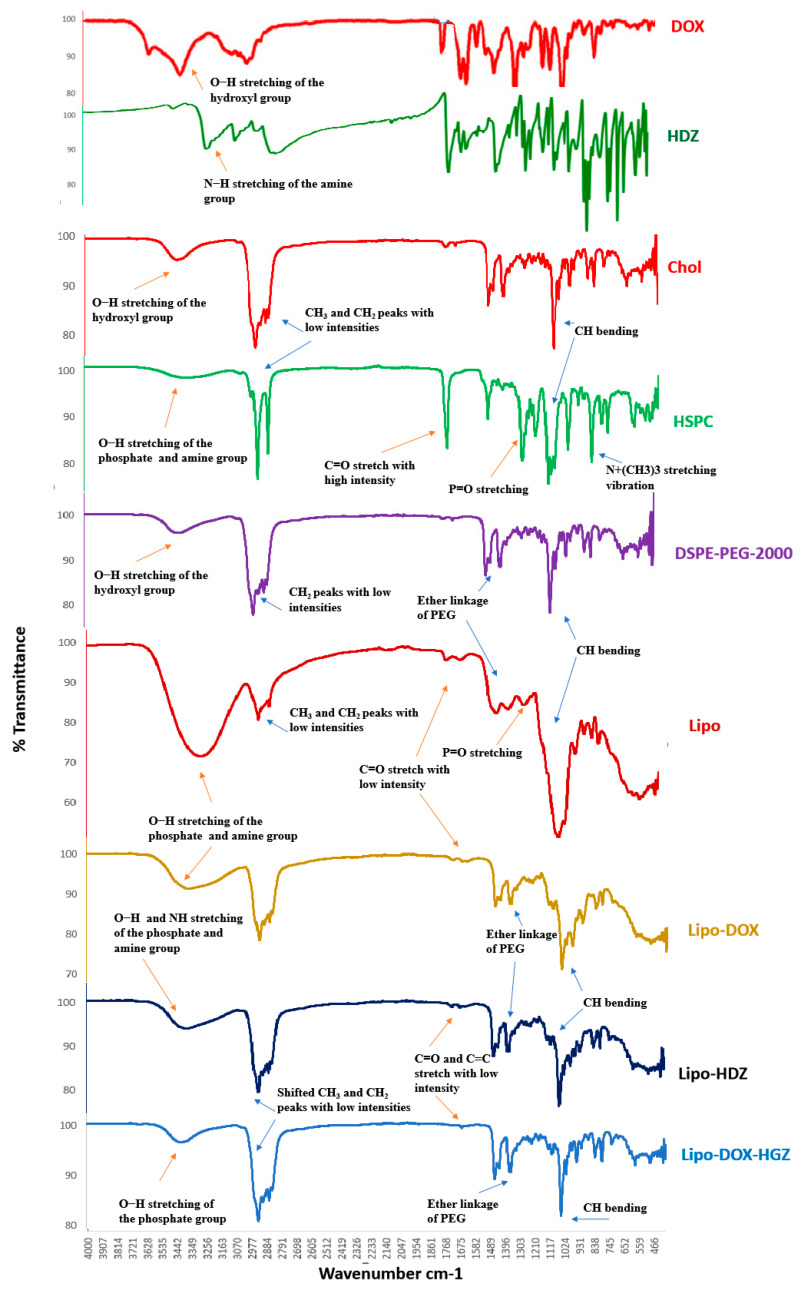
FTIR spectra of lyophilized liposomes encapsulating either DOX and HDZ together (Lipo-DOX-HDZ), only DOX (Lipo-DOX), or only HDZ (Lipo-HDZ) were compared to those of free liposomes and the liposomes’ precursors (HSPC, CHOL, and DSPE-PEG-2000).

The FTIR spectra of lyophilized liposomes encapsulating either DOX and HDZ together (Lipo-DOX-HDZ), only DOX (Lipo-DOX), or only HDZ (Lipo-HDZ) were compared to those of free liposomes, and the liposomes’ precursors (HSPC, CHOL, and DSPE-PEG-2000) were analyzed utilizing FTIR spectroscopy (Figure 5). In the HSPC, the characteristic C-H stretching vibrations and bending vibration of long-chain fatty acids at 2918 cm^−1^, 2851 cm^−1^, and 1468 cm^−1^, C=O stretching vibration at 1741 cm^−1^, P=O stretching vibration at 1234 cm^−1^, P-O-C stretching vibration at 1065 cm^−1^, and N+(CH_3_)_3_ stretching vibration at 722 cm^−1^ were observed [40]. The IR spectrum of pure cholesterol shows characteristics peaks at 3419 cm^−1^ (aliphatic O-H stretching) and 2934 cm^−1^ (CH asymmetric stretching of CH_3_) [41]. For DSPE-PEG 2000, the characteristic absorption bands are intense bands due to the associated hydroxyl groups (3421 cm^−1^), along with an intense band due to the ether linkage (C–O–C group; 1112 cm^−1^) [42].

Several changes were observed in the liposome formulations compared to the free liposome precursors: the chemical shift of P=O and P-O absorption to 1167 cm^−1^ and 1086 cm^−1^, respectively, and the high stretching C=O peaks converted to low intensity in the liposomes. However, the C-H characteristic peaks of long-chain fatty acids of phospholipids (2919 cm^−1^, 2851 cm^−1^, 1468 cm^−1^) still existed. Moreover, the hydroxyl peaks exhibited changes in position and intensity at 3480 cm^−1^ and 3350 cm^−1^, accounting for the interaction of DOX’s polar OH groups with the polar groups of HSPC and CHOL.

All of the liposome spectra exhibited a notable broad band between 3700 and 3100 cm^−1^, corresponding to O-H stretching of phosphate and cholesterol phenol groups, whereas HDZ exhibited a N-H band in the same region. Moreover, medium bands were observed at 2857 and 2927 cm^−1^ for CH_3_ and CH_2_, respectively, supporting the presence of intermolecular interactions between phospholipid hydrocarbon chains via van der Waals interactions [43]. In addition, all spectra showed a 1169 cm^−1^ band corresponding to the ether linkage of PEG groups. All spectra showed low-intensity stretching bands for C=O and C=C that could be attributed to the aromatic and carbonyl groups of the encapsulated drugs. Peak variation in the FTIR range may have been the effect of the formation of weak hydrogen bonds. Furthermore, the high similarities between all of the liposome spectra suggest good liposome integrity and stability before and after lyophilization, while simultaneously confirming the remote loading of DOX and HDZ into the aqueous core of the liposomes.

### 2.4. Cellular Uptake

#### 2.4.1. Flow Cytometry Analysis

**Figure 6:**
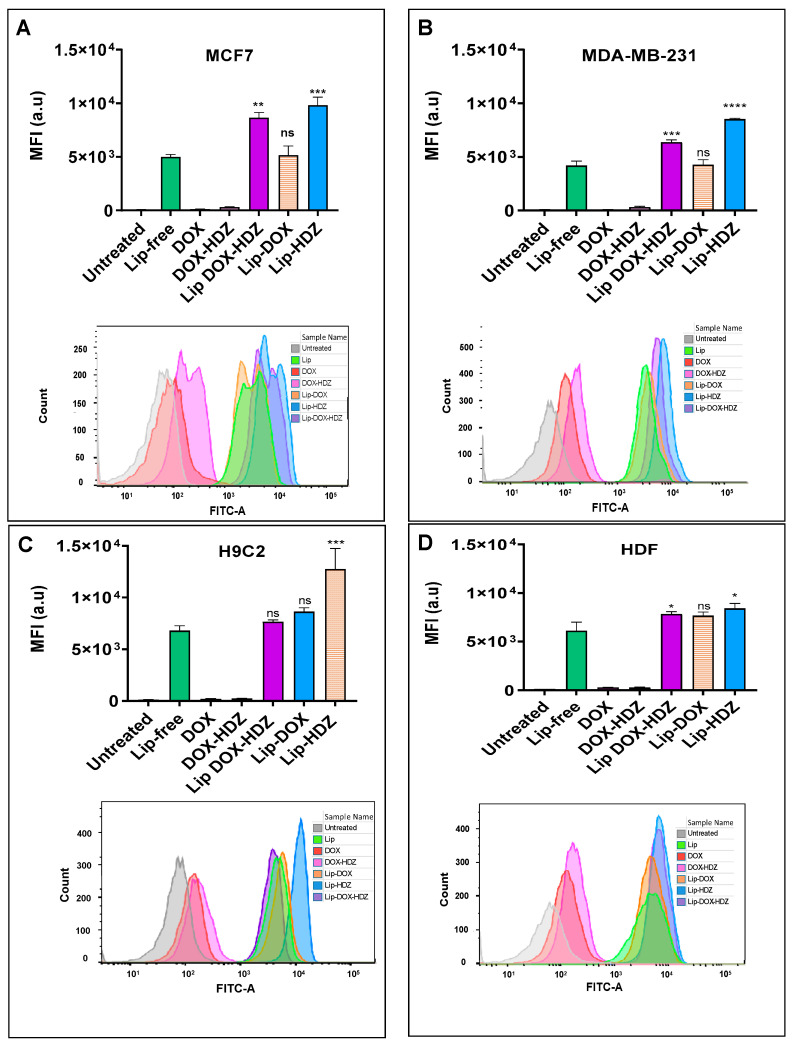
Flow cytometry analyses for cellular uptake of Lip, DOX, DOX-HDZ, Lip-DOX, Lip-HDZ, and Lip-DOX-HDZ in cultured cells compared to untreated cells for each cell line: (**A**) MCF7, (**B**) MDA-MB-231, (**C**) H9C2, and (**D**) HDF cells were treated with free DOX, DOX-HDZ, and Lip-DOX-HDZ and compared to untreated cells. Lip labeled with coumarin. * *p* < 0.05, ** *p* < 0.01, *** *p* < 0.001, **** *p* < 0.0001, ns: non-significant (one-way ANOVA). Data are presented as the mean ± SD (*n* = 3).

The internalization of drugs and liposomes into cells typically involves various processes, including passive diffusion, active transport, endocytosis, or receptor-mediated uptake, based on the characteristics of the drug molecules and the specific cell line [44,45]. Cellular uptake by four cell lines (MCF7, MDA-MB-231, HDF, and H2C9) was evaluated by measuring the mean fluorescence intensity (MFI) of coumarin-labeled liposomes (Figure 6). It was evident that all cell lines showed a detectable coumarin signal upon exposure to all liposomal formulations, confirming successful uptake compared to untreated cells. Notably, all cell lines exhibited a statistically significant increase (*p*-value < 0.05) in cellular uptake for Lip-HDZ, as well as for Lip-DOX-HDZ, in MCF7, MDA-MB-231, and HDF compared to Lip-Free. In contrast, Lip-DOX showed no significant changes in cellular uptake by all cell lines compared to Lip-free. The increased cellular uptake observed with Lip-HDZ and Lip-DOX-HDZ compared to Lip-free and Lip-DOX can be attributed to the drug–liposome interactions, which can affect the physicochemical properties and stability of HDZ-loaded liposomes (Lip-HDZ and Lip-DOX-HDZ).

These findings are in line with the results of Chen et al., who reported that the cellular uptake of PEG-DOX-LP increased by 5.1-fold compared to free liposomes [46]. Additionally, a study utilized a novel two-step solid-phase extraction method to separate liposomes from tissues and tumors accurately, enabling the characterization of drug release and uptake profiles of PEGylated liposomal doxorubicin in tumor-bearing mice. The results showed efficient release of doxorubicin into tumors, leading to a 1.8-fold increase in uptake compared to free drug administration, potentially reducing cardiotoxicity [47].

#### 2.4.2. Cellular Uptake: Confocal Laser Scanning Microscopy

Confocal microscopy is a powerful imaging technique that is commonly used in biology and medicine to obtain high-resolution images of biological samples. When studying drugs and drug liposomes using confocal microscopy, researchers are typically interested in visualizing the distribution and localization of these compounds within cells or tissues [48,49]. Confocal microscopy can provide insights into how liposomes are taken up by cells—whether they accumulate in specific organelles or are distributed throughout the cytoplasm [50].

**Figure 7:**
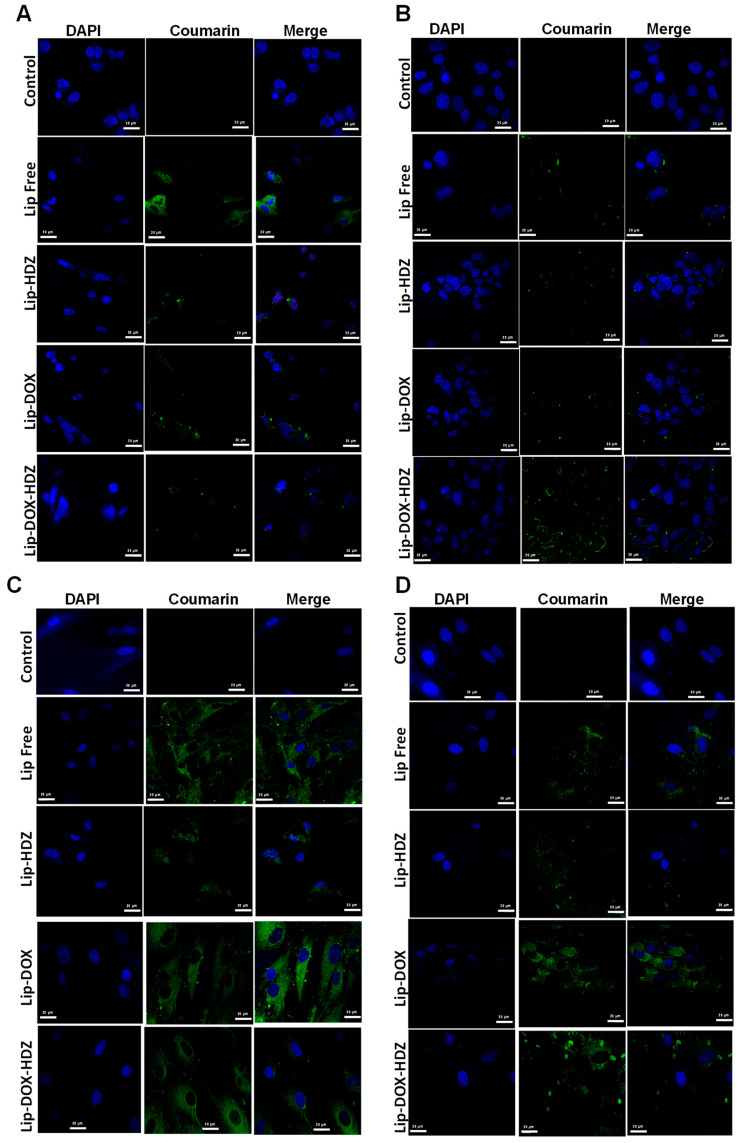
Confocal microscopy for each cultured cell line: (**A**) MDA-MB-231, (**B**) MCF7, (**C**) HDF, and (**D**) H2C9 cells were treated with free DOX, DOX-HDZ, and Lip-DOX-HDZ and compared to untreated cells. Lip labeled with coumarin.

To visualize the localization and accumulation of coumarin-labeled liposomes following uptake, the MCF7, MDA-MB-231, H9C2, and HDF cell lines were examined using confocal laser scanning microscopy (CLSM) (Figure 7). The CLSM images reveal that liposomal formulations exhibit predominant localization in the cytoplasm across all cell types, consistent with the quantitative analysis of cellular uptake.

In the current study, the cellular uptake determined by flow cytometry and confocal imaging cumulatively suggests that HDZ may enhance liposomal internalization. This pattern was maintained in multiple cell lines analyzed through flow cytometry, while confocal microscopy provided additional evidence to suggest that the predominant localization of liposomes is the cytoplasm. While Lip-HDZ alone had an upsurge in uptake by cancer cells, DOX-bearing Lip-DOX-HDZ seemed to behave differently as compared to Lip-DOX, which indicates that there is some form of interaction between HDZ and the liposome structure that facilitates easier penetration at cellular membranes. These observations stress the need to study the interplay of drugs and liposomes more closely to understand how to optimize the delivery of drugs using liposomes.

### 2.5. Cell Viability Assay

**Figure 8:**
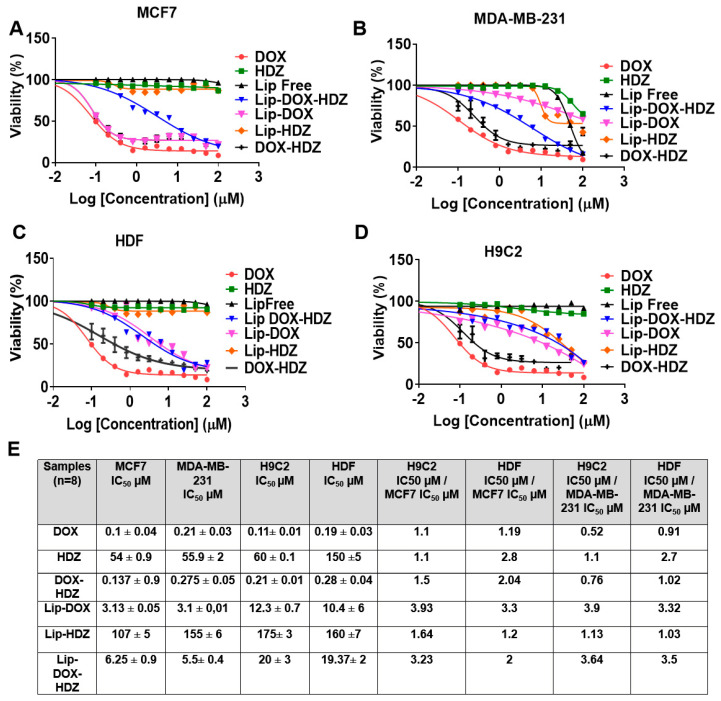
Cell viability assay of both breast cancer cell lines—(**A**) MDA-MB-231 and (**B**) MCF-7—(**C**) H9C2 normal cardiac cells, and (**D**) human dermal fibroblasts when treated with free DOX, HDZ, DOX-HDZ, Lip-DOX, Lip-HDZ, and Lip-DOX-HDZ at different concentrations for 72 h and 3 h. (**E**) Summary of IC50 and selectivity index values for the four cell types. Data are presented as the mean ± SD (*n* = 3).

The in vitro cytotoxicity assay on MDA-MB-231 revealed that that the IC_50_ values for the drugs were found to be 0.1 ± 0.04 µM for DOX, 54 ± 0.9 µM for hydralazine, and 0.13 ± 0.9 µM for the combination of both, whereas for Lip-DOX, Lip-HDZ, and Lip-DOX-HDZ they were 3.13 ± 0.05 µM, 107 ± 5 µM, and 6.25 ± 0.9 µM, respectively. These numbers also closely corresponded to the IC50 values observed in MCF7 cells. The toxicity of these drugs was observed to be 2 to 3 times lower when tested on cardiac cells and fibroblasts, as indicated by the selectivity values provided in Figure 8A–E.

### 2.6. Apoptosis by Flow Cytometry

Apoptosis, often referred to as programmed cell death, is a critical process maintaining the balance of cell proliferation and cell death within an organism. Dysregulation of apoptosis can lead to various diseases, including cancer [51]. DOX has been studied extensively for its effect on apoptosis in both cancer cell lines and normal cells, including cardiac cells [36,52]. Studies have also shown that hydralazine can induce apoptosis in cancer cell lines by reversing the hypermethylation of specific genes involved in apoptosis regulation and cell-cycle control [29,53].

**Figure 9:**
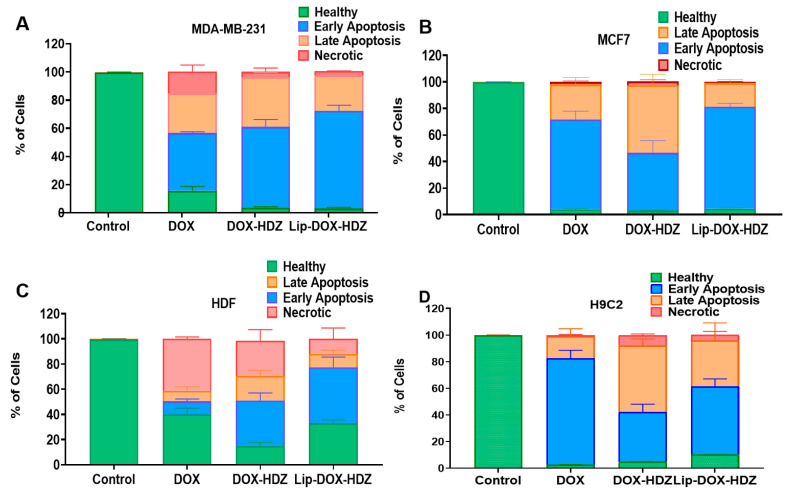
Apoptosis induced by DOX, DOX-HDZ, and Lip-DOX-HDZ treatment: (**A**) MDA-231, (**B**) MCF-7, (**C**) HDFs, and (**D**) H9C2. Data are presented as the mean ± SD (*n* = 3).

The effect of DOX and HDZ co-encapsulated into liposomes on the mechanism of cell death was examined using an apoptosis assay. In the MDA-23 and MCF7 breast cancer cell lines and HDF normal cells, Lip-DOX-HYD induced a slight increase in early apoptosis along with a decrease in necrosis, while in H9C2 cardiac cells there was an increase in late apoptosis and necrosis with the DOX-HDZ free drug combination and Lip-DOX-HDZ compared to free DOX (Figure 9 and Figure S1).

These results may be explained by the primary mechanism of action of hydralazine, which involves blocking the IP3-dependent release of calcium ions (Ca^2+^) from the sarcoplasmic reticulum (SR) [54]. Calcium signaling plays a crucial role in regulating various cellular processes, including apoptosis, and any imbalance can induce SR stress, leading to the activation of the unfolded protein response (UPR). Persistent SR stress can trigger apoptotic pathways, contributing to cell death. Calcium can also directly activate certain apoptotic proteins, such as calcineurin, which promotes apoptosis by dephosphorylating pro-apoptotic proteins or activating downstream apoptotic effectors [55,56].

### 2.7. Cell-Cycle Analysis by Flow Cytometry

**Figure 10:**
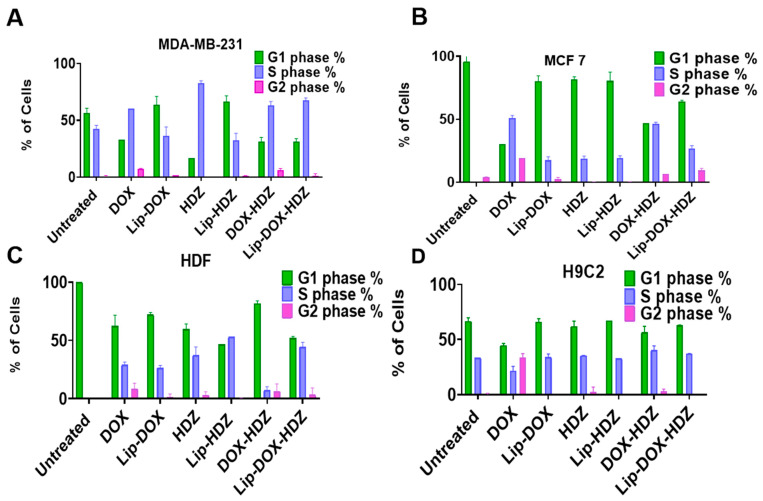
Cell-cycle analysis induced by DOX, DOX-HDZ, and Lip-DOX-HDZ treatment: (**A**) MDA-231, (**B**) MCF-7, (**C**) HDFs, and (**D**) H9C2. Data are presented as the mean ± SD (*n* = 3).

The cell cycle is a complex process through which cells replicate and divide. It consists of several distinct phases: G1 (gap 1), S (synthesis), G2 (gap 2), and M (mitosis). Various regulatory checkpoints ensure that each phase is completed accurately before progressing to the next phase [57]. We assessed the combined effect of DOX and HDZ on cell-cycle arrest (Figure 10 and Figure S2). It has been reported that doxorubicin significantly increased the G2 phase [58]. The effect of Lip-DOX-HDZ on the cell cycle showed significant cell-cycle arrest in the G1/S phase, with an effect on G2 compared to untreated cells. It was reported that DOX leads to cell-cycle arrest at various checkpoints, particularly the G1/S and G2/M transitions [59]. This allows the cell to repair DNA damage before progressing further through the cell cycle [60].

## 3. Materials and Methods

Hydrogenated soy phosphatidylcholine (HSPC) and cholesterol (CHO) were obtained from Carbosynth (Compton, UK). 1,2-Distearoyl-sn-glycero-3-phosphoethanolamine-N- [amino (polyethylene glycol)-2000] (DSPE-PEG ammonium salt) was obtained from Avanti Polar Lipids, Inc. (Alabaster, AL, USA). Doxorubicin and coumarin 6 (purity: 98%) were obtained from Sigma Aldrich (St. Louis, MO, USA).Hydralazine (purity > 99%) was obtained from TCI (Tokyo, Japan). Ethanol (purity: 99%) was obtained from Tedia (Fairfield, OH 45014, USA). Other solvents and chemicals were obtained from different sources.

### 3.1. Liposomal Formulation

Liposomes were formulated using ethanol injection with lipids. HSPC and DSPE-PEG 2000 were obtained from Avanti Polar Lipids, Inc. (Alabaster, AL, USA), and cholesterol (CHO) was obtained from Carbosynth (UK). Total lipids of 15.85 mg, composed of HSPC, cholesterol, and DSPE-PEG2000 in a 60:35:5 molar ratio, were dissolved in 200 µL of absolute ethanol. The dissolved lipids were slowly injected into 0.8 mL of 250 mM ammonium sulfate buffer (pH 3.5) at a 0.5 mL/min rate. The formed liposome suspension was stirred for 30 min at 600 rpm while maintaining the temperature at 65 °C. Downsizing of the liposomes was performed by extruding the liposomes for 13 cycles through a polycarbonate membrane (100 nm, Whatman^®^, Buckinghamshire, UK), using a Mini-Extruder (Avanti Polar Lipids, Inc. Alabaster, AL, USA), followed by dialysis tubing (12.5 KDa cutoff) in PBS (pH 6.5) containing 10% sucrose to replace the external ammonium sulfate buffer. In the active drug loading step, 1 mg each of DOX and HDZ was dissolved in PBS (pH 6.5) containing 10% sucrose at 60 °C with stirring. After that, the liposomes were cooled to a temperature of 2 to 8 °C and incubated for an hour. After that, the untapped DOX and HDZ were removed and washed two times with PBS solution using ultrafiltration with a 100 kDa cutoff Amicon filter (Millipore, Burlington, MA, USA). The encapsulated liposomes were stored at 4 °C.

### 3.2. Liposomal Characterization

#### 3.2.1. Measurement of Liposomes’ Hydrodynamic Diameter and Zeta Potential

Liposome samples (20 µL, ~0.3 mg of lipids) were diluted in 1 mL of deionized water. Then, the average hydrodynamic diameter, zeta potential, and polydispersity index (PDI) of the liposomes were assessed using Dynamic Light Scattering (DLS) with a Nano ZS instrument from Malvern Instruments (Worcestershire, UK). Data are expressed as the mean ± SD from at least 3 independent experiments.

#### 3.2.2. Liposomal Morphology Analysis by Transmission Electron Microscopy (TEM)

The morphology of blank liposomes, Lip-DOX, and Lip-HDZ was analyzed by transmission electron microscopy (TEM). Initially, Formvar-coated copper grids of 200 mesh (SPI Supplies, West Chester, PA, USA) and an ACE200 coating system (Leica, Vienna, Austria) were used. Subsequently, samples were coated by using 1.5% vinyl K in chloroform solution. Approximately 10 μL of liposomes was applied onto each copper grid and air-dried overnight. The dried grids were stained using a uranyl acetate dye solution, rinsed with distilled water, and air-dried. Finally, the grids were observed using a Versa 3D transmission electron microscope operated at 30 kV (FEI, Eindhoven, The Netherlands) [61].

#### 3.2.3. Evaluation of Encapsulation and Loading Efficiencies of Hydralazine and Doxorubicin into Liposomes

HDZ measurement was performed using an HPLC system (DIONEX TM Ultimate TM 3000, Waltham, MA, USA, Thermo Fisher) equipped with a reverse-phase C18 column (150 × 4.6 mm, 5 μm, KNAUER). The analysis was carried out under isocratic elution using a mobile phase consisting of ethanol and 0.1% trifluoroacetic acid at a ratio of 60:40% *v*/*v*. Chromatography was performed under isocratic conditions at a 1 mL/min flow rate at room temperature, and UV detection was performed at 270 nm. Each sample injection used 20 μL, and a calibration curve was established using standard HDZ solutions ranging from 0 to 0.2 mg/mL. Moreover, the quantity of DOX loaded into the liposomes was measured based on the fluorescence intensity of doxorubicin, using a Glomax microplate reader (Promega, Madison, WI, USA). The excitation peak was measured at 490 nm, along with an emission peak at 510–570 nm. To quantify cholesterol (CHO), a mobile phase consisting of 60% isopropanol, 30% acetonitrile, and 10% water with 0.02% formic acid was used, with similar chromatographic conditions to HDZ and standard CHO concentrations ranging from 0 to 1.6 mg/mL.

Next, 200 μL of liposomes was disrupted using 800 μL of acetonitrile, followed by HPLC analysis. The encapsulation efficiency (EE%) and loading efficiency (LE%) of the drugs were calculated using the following equations:

EE (%) = ([Drug trapped in liposomes])/([Amount drug added]) × 100(1)

LE (%) = ([Drug trapped in liposomes])/([Amount of lipids and drug]) × 100(2)

#### 3.2.4. Release of DOX and HDZ from Liposomes

The release of DOX and HDZ from the liposomes was assessed under various conditions at 37 °C and 4 °C in PBS (pH 7.4), over a 14-day period. At each timepoint, the mean hydrodynamic diameter and polydispersity index (PDI) of the liposomes were measured using previously established methods. Additionally, the release of 1 mg/mL each of DOX, HDZ, Lip-DOX, Lip-HDZ, and Lip-DOX-HDZ was evaluated in 30 mL of PBS at 37 °C in different time intervals. The liposomes were prepared with drug-to-total-lipids (*w*/*w*) ratios of 0.01 for Lip-DOX, 0.05 for Lip-HDZ, and 0.042 for DOX and 0.025 for HDZ in Lip-DOX-HDZ (data obtained from EE%). At each timepoint, free DOX and HDZ were separated from the liposomal fraction using ultrafiltration with a 100 kDa dialysis bag^®^ (Millipore, Burlington, MA, USA). The DOX concentrations in the liposomal fraction were quantified using Glomax, while HDZ release was measured by HPLC, following previously described methods. The released amounts of DOX and HDZ were calculated based on the following equation:

Release% = (Initial concentration-concentration at time)/(Initial concentration) × 100(3)

#### 3.2.5. Lyophilization of Liposomal Formulations

Following liposome preparation, the liposomes were kept at −70 °C for 24 h, underwent freeze-drying for an additional 24 h, and were subsequently refrigerated for one week at 4 °C. The liposomes were then reconstituted in deionized water, evaluated for stability using DLS, and scanned for their full IR spectrum.

#### 3.2.6. Fourier-Transform Infrared (FTIR) Spectroscopy

Fourier-transform-infrared (FTIR) spectroscopy for the characterization of the lyophilized liposomes was performed using a Perkin-Elmer Spectrum Two with a universal ATR FTIR Spectrometer over a spectral range of 4000 to 400 cm^−1^. The FTIR spectra of lyophilized liposomes encapsulating either DOX and HDZ together (Lipo-DOX-HDZ), only DOX (Lipo-DOX), or only HDZ (Lipo-HDZ) were compared to those of free liposomes and the liposomes’ precursors (HSPC, CHOL, and DSPE-PEG-2000) to evaluate their interactions.

#### 3.2.7. Thermogravimetric Analysis (TGA) and Differential Scanning Calorimetry (DSC)

The weight loss of free liposomes, Lip-DOX, Lip-HDZ, and Lipo-DOX-HDZ was analyzed using a Shimadzu DSC instrument (Kyoto, Japan). The DSC patterns were obtained by heating the liposomes to 50 °C and then cooling them with liquid nitrogen to −50 °C. The temperature was gradually increased at a rate of 5 °C per minute under a constant flow of nitrogen gas (100 mL/min). The thermal performance, decomposition rate, and weight loss percentage of Lip-DOX, Lip-HDZ, and Lipo-DOX-HDZ compared to free liposomes, DOX, and HDZ were determined using TGA. This analysis was performed with a Mettler Toledo instrument (TGA/DSC 2, Greifensee, Switzerland) from 25 °C to 600 °C, at a heating rate of 10 °C per minute. Alumina crucibles (1–2 mg of each sample) were used.

### 3.3. Cell Culture

Breast cancer cell lines (MDA-MB-231 and MCF7) and normal cell lines (rat cardiac cells (H9C2) and human dermal fibroblasts (HDFs)) were obtained from the American Type Culture Collection (ATCC, Manassas, VA, USA). MCF7 cells were cultured in RPMI 1640 medium (EuroClone, Milan, Italy), and the other three cell lines were cultured in DMEM high glucose. Both types of media were supplemented with 1% penicillin–streptomycin (EuroClone, Italy), heat-inactivated fetal bovine serum (FBS) (EuroClone, Italy), and L-glutamine. Cells were incubated in a 5% CO_2_ incubator (Memmert, Schwabach, Germany).

#### 3.3.1. Cellular Uptake Evaluation

Cellular uptake in four cell lines (MCF7, MDA-MB-231, HDFs, and H9C2) was evaluated using a BD FACSCanto™ II flow cytometer with BD FACSDiva™ software version 8.0 (BD, Franklin Lakes, NJ, USA). Approximately 100,000 cells of each cell line were seeded in 12-well plates and incubated for 24 h at 37 °C. After incubation, the cells were washed with PBS and incubated in fresh medium. Then, 100 μL of coumarin-labeled free Lip, Lip-DOX (DOX = 0.01 wt/wt), Lip-HDZ (HDZ = 0.05 wt/wt), and Lip-DOX-HDZ (DOX = 0.42 wt/wt and HDZ = 0.025 wt/wt) was added and incubated with the cells for 4 h at 37 °C. Following incubation, the cells were detached and transferred into flow cytometry tubes, and the mean fluorescence intensity (MFI) was measured in 10,000 events. Untreated cells and cells treated with coumarin-labeled free Lip were used as controls for comparison.

#### 3.3.2. Confocal Laser Scanning Microscopy (CLSM)

To visualize cellular uptake, MDA-MB-231, MCF7, HDF, and H2C9 cells (100,000 cells each) were seeded onto glass coverslips in 12-well plates (SPL, Gyeongbuk, Republic of Korea) and incubated at 37 °C for 24 h. Following incubation, the cells were washed with PBS and supplemented with 1 mL of fresh medium. Subsequently, 100 μL of coumarin-labeled Lip formulations was added in triplicate and incubated with the cells at 37 °C for 4 h. After incubation, the cells were washed twice with PBS. Then, 1 mL of 4% formaldehyde was added and incubated for 15 min at room temperature in the dark. Post-incubation, the cells were washed twice with PBS. The coverslips were mounted onto glass slides in medium with 4′,6-diamidino-2-phenylindole dihydrochloride (DAPI). Then, all slides were investigated using a Zeiss LSM780 confocal microscope system (Carl Zeiss AG, Baden-Württemberg, Germany) [62].

#### 3.3.3. Assessing Cell Viability by MTT Assay

To measure the cytotoxicity of the prepared formulation against the MDA-MB-231, MCF7, HDF, and H9C2 cell lines, cells were seeded into a 96-well plate (Corning, NY, USA) at a density of 9000 cells per well. The cells were treated with varying concentrations of DOX, HDZ, and their liposomes, ranging from 0.001 to 100 μM, and incubated at 37 °C with 5% CO_2_ for 72 h. After incubation, the old medium was replaced with 100 μL of fresh medium containing MTT assay salt (Bioworld, Visalia, CA, USA) in each well. The plates were then incubated again at 37 °C for 3 h. DMSO (50 μL) was added to each well to dissolve the formazan crystals. Absorbance was measured at 560 nm using a Glomax plate reader (Promega, Madison, WI, USA) to assess cell viability. The IC50 values of HDZ (from TCI, Tokyo, Japan) and DOX were determined using an MTT assay (Fisher Scientific, USA).

Cell Viability% = [Sample absorbance mean]/[Control Absorbance mean] × 100 (4)

#### 3.3.4. Exploring Apoptosis of Cells Treated with Lip-DOX-HDZ

The growth inhibition of cells treated with HDZ, DOX, DOX-HDZ, and their liposomes was investigated by determining the mechanism of cell death using annexin V/propidium iodide (PI) staining and flow cytometry. Cells were seeded into 12-well plates at 100,000 cells per well and exposed to free DOX (0.5 μM), the DOX-HDZ free drug combination (equivalent to 0.5 μM DOX and 50 μM HDZ), and Lip-DIX-HDZ (equivalent to 0.5 μM DOX and 50 μM HDZ) and incubated for 24 h. After incubation, the cells were detached using Accutase enzyme (StemPro™, Gibco™, Leicestershire, UK), washed with PBS, and stained using an annexin V/PI apoptosis kit (R&D Systems, Minneapolis, MN, USA), following the kit’s instructions. A total of 10,000 counts were calculated with a BD FACS CANTO II and analyzed using BD FACS Diva™ software version 7.0.

#### 3.3.5. Investigating Cell-Cycle Control of Cells Treated with Lip-DOX-HDZ

The DNA content of the cells was investigated using flow cytometry; each cell line was seeded in 6-well plates at a density of 5 × 10^5^ cells per well and treated with 0.5 μM DOX, 50 μM HDZ, 0.5 μM DOX-HDZ, and their liposomes. After one day, the cells were trypsinized with cell dissociation reagent (Accutase enzyme, StemPro™, Gibco™, Leicestershire, UK) and washed with PBS. Cold (−20 °C) 70% ethanol was gradually added, and the cells were incubated on ice for 10–15 min. After incubation, the cells were washed twice with PBS by centrifugation and rehydrated in PBS for 10 min. The cell pellet was then resuspended in 300 µL of staining buffer and propidium iodide (PI) solution (1 mg/mL) (Invitrogen, Carlsbad, CA, USA), followed by a 1-hour incubation in the dark at room temperature. A total of 10,000 events were counted using a BD FACS CANTO II and examined with BD FACS Diva™ software version 7.0.

## 4. Conclusions

The current work describes PEGylated liposomes successfully co-loaded with doxorubicin and hydralazine (Lip-DOX-HDZ) using a remote pH gradient loading method. Comprehensive characterization of Lip-DOX-HDZ via TEM, DLS, FTIR, TGA, and DSC confirmed the nanosized Lip-DOX-HDZ liposomes (~150 nm) with low polydispersity, structural integrity, and stability. Notably, Lip-DOX-HDZ exhibited efficient cellular uptake and localization. The cytotoxic activity of Lip-DOX-HDZ was assessed in two breast cancer cell lines (MCF7 and MDA-MB-231) and was further compared to that in normal cell lines (human dermal fibroblasts (HDFs) and rat cardiac cells (H9C2)). Our findings showed lower toxicity in HDF and H9C2 normal cells compared to MCF7 and MDA-MB-231 breast cancer cells. These findings highlight the potential of Lip-DOX-HDZ as a stable and effective nanocarrier of doxorubicin and hydralazine into cancer cells. Moreover, successful remote co-loading represents a versatile approach that can be applied to different drug combinations to improve therapeutic outcomes.

## Figures and Tables

**Table 1:** Summary of average hydrodynamic diameter (z-average size), zeta potential, encapsulation efficiency (EE%), and loading efficiencies (LE%). Data are presented as the mean ± SD (*n* = 3).

Liposomes (*n* = 3)	Hydrodynamic Diameter (d. nm)	Polydispersity Index (PDI)	Z-Potential (mV)	Encapsulation Efficiency (%)	Drug Loading (%)
Free Lip	150 ± 9	0.09 ± 0.06	−17 ± 2	-	-
Lip-HDZ	157 ± 12	0.12 ± 0.06	−32 ± 2	42 ± 0.4	2.6
Lip-DOX	155 ± 18	0.18 ± 0.08	−33 ± 5	90 ± 8.3	5.7
Lip-DOX-HDZ	158 ± 18	0.22 ± 0.08	−22 ± 5	DOX: 90 ± 7.7	5.7
				HDZ: 21 ± 1.7	1.3

## Data Availability

The original contributions presented in this study are included in the article/Supplementary Material. Further inquiries can be directed to the corresponding author(s).

## Supplementary Materials

The following supporting information can be downloaded at https://www.mdpi.com/article/10.3390/molecules30071549/s1: Figure S1: Apoptosis dot-plot graphs for treated cells; Figure S2: Cell-cycle histograms for treated cells.
