# Supplementary material for: Remote Co-Loading of Doxorubicin and Hydralazine into PEGylated Liposomes: In Vitro Anti-Proliferative Effect Against Breast Cancer
Source: Molecules. 2025 Mar 31;30(7):1549. doi: 10.3390/molecules30071549 (PMC11990610; doi:10.3390/molecules30071549)
Supplement: Supplementary file 1 [file molecules-30-01549-s001.zip › molecules-3414852-supplementary.pdf]

Supplementary Materials

Remote Co-Loading of Doxorubicin and Hydralazine into PEGylated Liposomes: In Vitro Anti-Proliferative Effect Against Breast Cancer

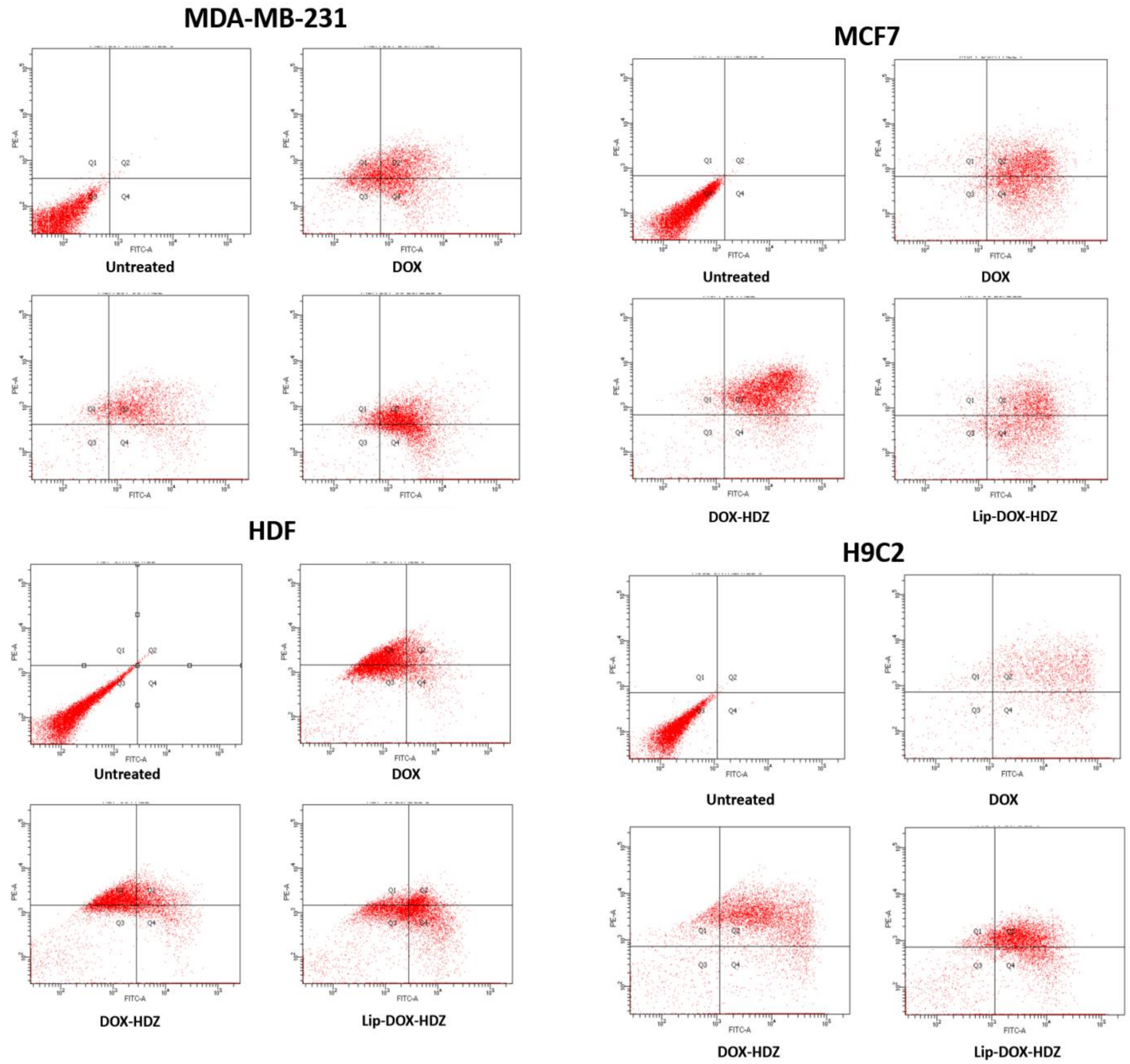

Figure S1: apoptosis dot-plot graphs for treated cells.

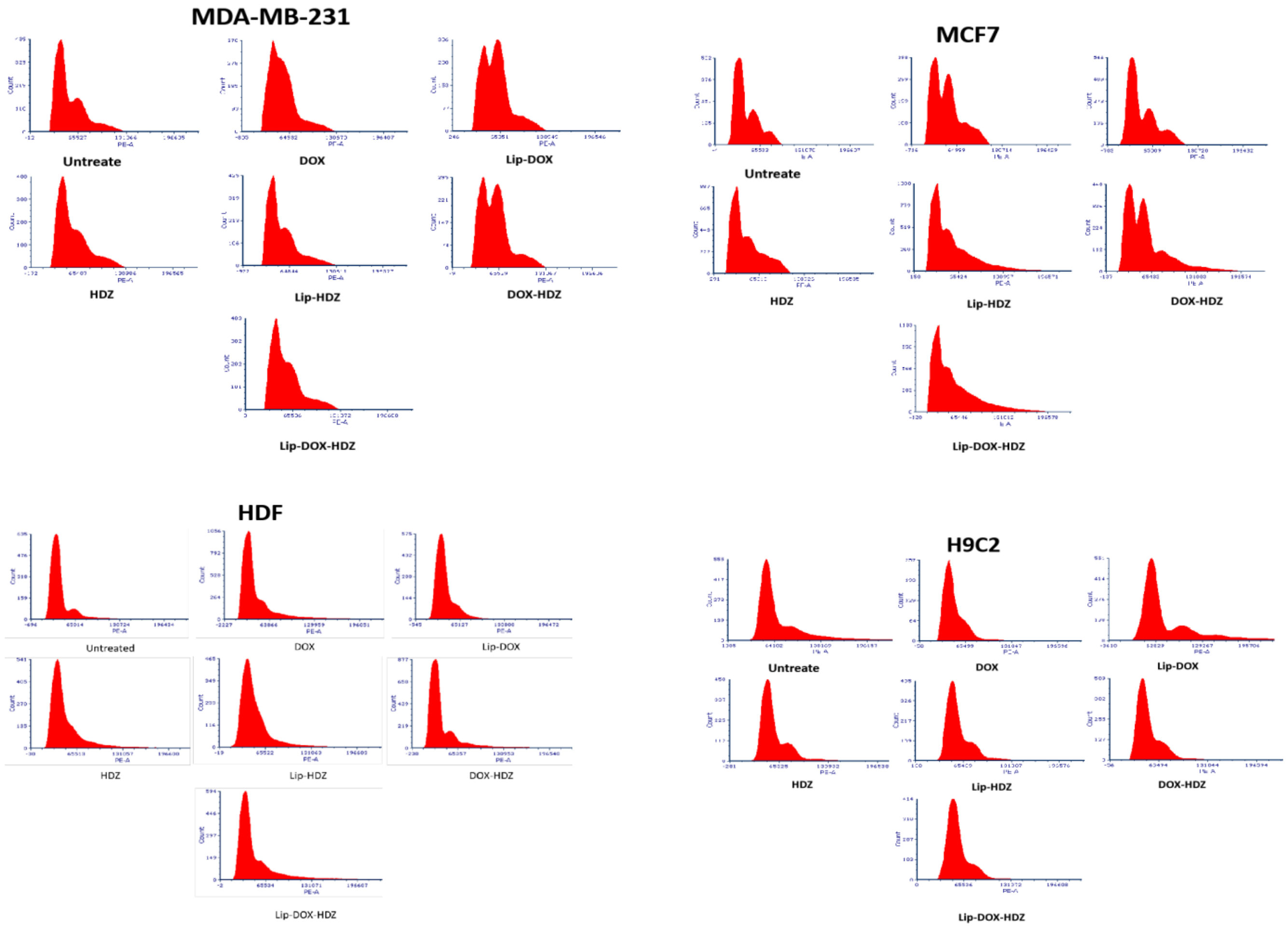

Figure S2: Cell cycle histograms for treated cells.
